# Can Nutraceuticals Support the Treatment of MASLD/MASH, and thus Affect the Process of Liver Fibrosis?

**DOI:** 10.3390/ijms25105238

**Published:** 2024-05-11

**Authors:** Aneta Sokal-Dembowska, Sara Jarmakiewicz-Czaja, Katarzyna Ferenc, Rafał Filip

**Affiliations:** 1 Institute of Health Sciences, Medical College, Rzeszow University, 35-959 Rzeszow, Poland; 2 Institute of Medicine, Medical College, Rzeszow University, 35-959 Rzeszow, Poland; 3 Department of Gastroenterology with IBD Unit, Clinical Hospital No. 2, 35-301 Rzeszow, Poland

**Keywords:** antioxidants, beta-carotene, curcumin, liver fibrosis, nutraceuticals, omega-3 fatty acids

## Abstract

Currently, metabolic dysfunction-associated steatotic liver disease (MASLD) and metabolic dysfunction-associated steatohepatitis (MASH) are considered to be the main causes of fibrosis. In turn, fibrosis may lead to the development of hepatocellular carcinoma or advanced cirrhosis, i.e., potentially life-threatening conditions. It is likely that therapy aimed at reducing the risk of developing hepatic steatosis and inflammation could be helpful in minimizing the threat/probability of organ fibrosis. In recent years, increasing attention has been paid to the influence of nutraceuticals in the prevention and treatment of liver diseases. Therefore, the aim of this review was to describe the precise role of selected ingredients such as vitamin C, beta-carotene, omega-3 fatty acids, and curcumin. It is likely that the use of these ingredients in the treatment of patients with MASLD/MASH, along with behavioral and pharmacological therapy, may have a beneficial effect on combating inflammation, reducing oxidative stress, and thereby preventing liver damage.

## 1. Introduction

The incidence of liver fibrosis is now closely related to obesity, and the main causes are considered to be metabolic dysfunction-associated steatotic liver disease (MASLD) and metabolic dysfunction-associated steatohepatitis (MASH), rather than previously observed viral infections [1]. Fibrosis can lead to the development of hepatocellular carcinoma or advanced cirrhosis [2]. It is also considered a key predictor of liver diseases that cause the cirrhosis of the liver [3]. Fibrinogenesis is a dynamic process that leads to the activation of liver myofibroblasts and the excessive accumulation of extracellular matrix (ECM) [4]. Interestingly, approximately 33% of the patients diagnosed with MASH are believed to develop liver fibrosis, while cirrhosis may develop in around 20% of the cases [5]. Therefore, in order to minimize the risk of liver fibrosis, therapy aimed at reducing the risk of developing steatosis and hepatitis seems to be important [6]. Zhang et al. highlighted the role of medical treatment in liver fibrosis in a recent review. According to the authors, antifibrotic treatment should include behavioral therapy, medications, and nutritional factors. Introducing such a procedure may be helpful in reducing inflammation and oxidative stress and preventing the death of hepatocytes, which may significantly reduce the risk of fibrosis and organ failure [7].

Current guidelines for patients with liver conditions emphasize the importance of ensuring the adequate intake of calories, protein, and vitamin D. In turn, in the case of vitamin A and B vitamins, attention is drawn to the risk of their deficiency [8]. However, recent attention has also been directed towards the potential benefits of nutraceuticals in preventing and treating MASLD and MASH [9,10].

Nutraceuticals are foods that exhibit therapeutic and disease-preventing effects [11]. The nutraceuticals may aid in reducing chronic inflammation and oxidative stress, and protecting liver cells [12]. Therefore, the purpose of our review is to delve into the role of other compounds such as vitamin C, beta-carotene, omega-3 fatty acids, and curcumin in this context.

## 2. Selected Cells Involved in the Liver Fibrosis Process

The process of liver fibrosis is complex and depends on many factors. Damage to normal liver tissue results in the accumulation of ECM proteins, and this is mainly cross-linked interstitial collagen (types I, III, and VI), basement membrane collagen (type IV), and other proteins [13,14]. The liver cells (hepatocytes) themselves can be damaged by metabolic, toxic, or viral factors [15].

### 2.1. Hepatocytes and Hepatic Stellate Cells (HSC)

Hepatocytes, or liver cells, in the progression of MASH, can have their basic functions inhibited through changes in the transcriptome and genome. Degraded hepatocytes, through the release of fibrinogen mediators, activate stellate cells and recruit macrophages [16]. The activation of stellate cells and the progression of liver fibrosis are also influenced by hepatocyte pyroptosis, which is cell death by cell membrane rupture through the activation of caspase-1. Hepatocytes after pyroptosis release NLRP3 (NOD-, LRR- and pyrin domain-containing protein 3) inflammasome proteins, which promote fibrinogenesis [17]. Inflammasomes, the multiprotein complexes responsible for activating inflammatory processes, can be stimulated by stellate cells responsible for ECM formation and fibrosis progression. In addition, the pyroptosis of hepatocytes can be caused by releasing pro-inflammatory cytokines [18]. Other factors in the activation of stellate cells are NK cells (Natural Killer), B cells, and platelets [19]. Damage-associated molecular patterns (DAMPs) and especially “mito-DAMPs” (the structural elements of mitochondria) released as a result of damage to hepatocytes exhibit potent immunogenic effects. They may potentially be involved in liver fibrosis [20]. Transmembrane Notch receptors determine cell fate during development. In the presence of MASH, Notch activity in hepatocytes can be a biomarker of the severity of the disease. This is due to the response to Opn (osteopontin) secretion through the Notch signaling of hepatocytes, which in turn activates stellate cells [21]. Furthermore, Notch signaling can increase the infiltration of liver monocyte-derived macrophages, depending on MCP-1 (chemokine monocyte chemoattractant protein-1) [22].

Connective tissue growth factor (CTGF), transforming growth factor β (TGFβ), tumor necrosis factor (TNF), lipid peroxides, reactive oxygen species (ROS), and other cytokines and chemokines show the ability to activate signaling pathways to proliferate HSCs in migratory and contractile myofibroblasts. In turn, they secrete ECM molecules such as type I and type III collagen to form scar tissue. Furthermore, activated HSCs secrete Endothelin-1 (ET-1), which shows the ability to constrict blood vessels and lead to inflammatory processes [23,24,25]. Consequently, this contributes to the occurrence of various complications, such as portal hypertension [26]. Khomich et al. indicate that the metabolic reprogramming of the cell is required for the synthesis of ECM in HSCs. Understanding the mechanisms of metabolic changes that occur in cells can direct the development of a therapeutic intervention in liver fibrosis [27].

### 2.2. Liver Macrophages

Macrophages are among the phagocytic cells that are involved in the protective mechanisms of the body. Macrophages that reside in the liver are derived from monocytes and Kupffer cells (KCs) (sedimented macrophages found in the wall of the sinusoidal vessels of the liver; they account for 1/5 of all extrahepatic cells) [28]. These cells play an important role in wound healing and the initiation of the response to infection. Significantly, during liver injury, macrophages can interact with hepatocytes through, among other things, vesicles secreted by liver cells and through DAMPs (damage-associated molecular patterns) [29]. Some researchers indicate that KCs show the ability to initiate and progress MASH (metabolic dysfunction-associated steatohepatitis) [30,31]. During inflammation, both monocyte-derived macrophages and KCs show the expression of pro-inflammatory cytokines, i.e., TNFα (tumor necrosis factor alpha), IL-6 (interleukin 6), and chemokines, e.g., CCL2 (C-C motif chemokine ligand 2), which consequently exacerbate inflammation through the influx of macrophages from monocytes [14]. Activated macrophages secrete TGFβ1, which is a major cytokine that induces fibrinogenesis in activated myofibroblasts and stellate cells [32]. Macrophages, depending on various factors (pathogenic microorganisms, cytokines, etc.), show the ability to differentiate into different phenotypes. Such properties of macrophages are known as polarity. Researchers indicate that such macrophage polarization may exert an influence on the process of liver fibrosis, but due to the complex mechanism, they also indicate that more research is needed in this area [33,34]. Furthermore, Rao et al. present that FSTL1 (follistatin-like 1) macrophages may exhibit the ability to induce M1 polarization by reprogramming PKM2 (pyruvate kinase muscle isozyme M2) macrophages, thus exerting an effect on liver fibrosis progression [35]. Similarly, FGF12 (fibroblast growth factor 12) macrophages can also induce the progression of organ fibrosis [36].

### 2.3. Lymphocytes

Lymphocytes belonging to the cells of the immune system are also involved in the process of liver fibrosis. One of the groups involved in fibrinogenesis is CD8+ T lymphocytes, which are essential for the annihilation of tumors and infected cells. Although these cells can stimulate cell proliferation, they reduce the differentiation of liver progenitor cells into hepatocytes [37]. On the other hand, Bonilla et al. in their work indicate that this type of lymphocytes producing interferon gamma (IFNg) is associated with a low rate of the progression of liver fibrosis [38]. The fibrosis of the organ itself may contribute to the decreased immune surveillance of CD8+ T cells, which may consequently induce an inappropriate response toward infected cells [39]. Researchers describe CD4 + cells as cells that play conflicting roles in liver fibrinogenesis depending on the cell type (Th2 (T helper 2 cells) promotes fibrosis). The cells that are also involved in organ fibrosis are Treg (regulatory T cells) and Th17 (T helper 17 cells), and the relationship between them is also important [40,41]. Another group of cells are NK cells, and cells residing in the liver differ from peripheral blood NK cells in cytotoxicity and activation level, among other things. Liver NK cells exhibit inhibitory and fibrosis-promoting effects on the organ. They can promote profibrotic processes by increasing inflammation [42,43].

Other cell types are also known to produce liver myofibroblasts, e.g., bone marrow, portal fibroblasts, mesothelial cells, and platelets [44,45,46].

The interaction between immune cells and liver cells can regulate the progression of organ fibrosis, but the findings must be interpreted with caution to potentially exploit such interactions in the regression of liver fibrosis.

## 3. Regression of Liver Fibrosis

For quite a long time, liver fibrosis was considered an irreversible process [47]. However, recently, fibrosis has been observed to subside when the main causative factor in viral hepatitis, alcoholic liver disease, biliary obstruction, and obesity is eliminated [48]. As of now, it is known that fibrosis is reversible, and cirrhosis (i.e., impaired flow and liver architecture) can also be reversed in some cases [49]. The regression of cirrhosis has been observed in patients with copper and iron overload; alcohol abuse; hepatitis B, C, and D; secondary biliary cirrhosis; MASH; and autoimmune hepatitis [50]. The regression of liver fibrosis is illustrated by the reduced levels of fibrogenic and inflammatory cytokines, increased collagenase activity, and the atrophy of myofibroblasts and fibrous scarring [51]. Understanding the mechanisms of fibrosis and the focus of many researchers on this problem gives hope in the future for potentially approved treatments/drugs for patients with MASH. However, because the mechanisms of fibrosis are our natural physiological defense functions, the targets of treatment interventions and support for patients must be selected with great care [52].

Marcellin et al. showed that the long-term inhibition of hepatitis B virus (HBV) progression by tenofovir dizoproxil fumarate (DF) in patients with cirrhosis resulted in the regression of both fibrosis and cirrhosis [53]. Kong et al. inferred that in patients with HBV, changes in liver stiffness during the first 6 months of entecavir treatment may predict the potential reversal of liver fibrosis at month 18 of antiviral treatment [54]. In addition, D’Ambrosio et al. observed similar results in patients with hepatitis C virus (HCV) [55]. Sustained virologic response (SVR) induces benefits for the patient, regardless of the stage of fibrosis. SVR leads to an increased quality of life, reduced mortality, and reduced risk of complications that are associated with end-stage liver failure [56,57]. However, there are studies that show that despite achieving SVR, about 10% of the patients maintain fibrosis. Significantly, even progression to cirrhosis is found [58,59,60]. It is also worth mentioning that despite the favorable response to SVR, patients with cirrhosis should monitor their health for the development of hepatocellular carcinoma (HCC) for 8 to 10 years consecutively [61]. Fibrosis progression is found not only in viral diseases, but also in alcoholic steatohepatitis. Takahashi et al. presented the case of a 70-year-old Japanese patient who had consumed about 210 g of ethanol daily since he was 20 years old. The patient was diagnosed with alcoholic cirrhosis. After a period of passive abstinence due to stroke, the patient showed the regression of fibrosis [62]. In addition to excessive alcohol consumption, there is an obesity problem in society. As a result of the increased prevalence of obesity and the associated metabolic syndrome, MASH is considered one of the most common causes of chronic liver disease. Currently, there are no clear-cut guidelines for the treatment of MASH, which is why it has received much attention in recent years. Glass et al. selected a group of 45 patients diagnosed with MASH and subjected them to serial liver biopsies. This study was designed to control and monitor their clinical condition. As a result of this study, they showed that the regression of fibrosis is possible in patients with MASH, even at an advanced stage. Importantly, the only factor that influenced this was a loss of total body weight ≥10%. In addition, factors such as gender, age, carbohydrate disorders, and ferritin scores did not affect regression [63]. Vilar-Gomez et al. in a study involving 293 patients with MASH confirmed that the best results in the regression of liver fibrosis were obtained in patients with weight loss ≥10% [64]. In patients with MASH-induced cirrhosis, the regression of fibrosis has been shown to reduce complications from liver impairment [65]. In addition, it appears that regression is also possible in autoimmune hepatitis (AIH). Sun et al. showed that in AIH, corticosteroid treatment can halt the development and even reverse fibrosis. This appears to be the result of the inhibition of inflammatory activity [66]. Currently, the main treatment for AIH is the normalization of the biochemical markers of liver inflammation. Hartl et al. in a study involving 60 patients using biopsy and elastography found biochemical normalization to be a major predictor of favorable prognosis, but also of the potential regression of fibrosis [67]. On the other hand, Bardou-Jacquet et al. found the possibility of the regression of severe liver fibrosis in patients with hemochromatosis (C282Y mutation) as a result of the treatment process [68]. Sexual dysmorphism involving the liver has received attention for many years, but the mechanisms that differentiate the sexes are not fully understood. In an in vivo study, Sayaf et al. showed that male mice were more prone to develop severe fibrosis following acute liver injury. It seems that profibrogenic processes in the early stages of the disease are more prominent in the male sex, most likely by a mechanism of the intensive recruitment of neutrophils, among others [69]. Calvente et al. showed that mice with neutrophil depletion during hepatitis recovery show early fibrosis and changes in the liver architecture [70].

### Mechanisms of Regression

The successful regression of fibrosis requires the activation of several mechanisms. These include the removal/interruption of the causative agent, elimination or inactivation of myofibroblasts, inactivation of inflammation with the simultaneous activation of regenerative pathways, and degradation of the extracellular matrix [49,71].

Studies clearly indicate that the interruption of the agent as a result of viral eradication or suppression causes the interruption of the fibrosis process, but also affects the regression process [53,56,57,62]. The interruption of the causative factor causes pro-inflammatory pathways to switch to anti-inflammatory/regenerative pathways. Consequently, this promotes the regression of fibrosis [72].

A reduction in the number of active HSCs appears to be an important aspect of the regression of fibrosis [73]. Compared to active HSC, inactivated ones have a reduced expression of fibrogenic genes [74]. Puche’a et al. showed that reducing the number of HSCs in mice with induced liver fibrosis resulted in attenuated fibrosis and liver damage [75]. HSC reduction can occur through several processes. These include apoptosis, the aging process, but also a return to an inactivated phenotype [73]. Apoptosis, or programmed cell death physiologically, is responsible for regulating the balance between dying and proliferating HSCs during the fibrogenic process. As a result, apoptosis affects the reduction of myofibroblasts. Despite this, it is not sufficient to restore liver integrity [71]. Recently, scientists have turned their attention to a mechanism called mitophagy. Mitophagy is a mechanism that is responsible for the elimination of damaged mitochondria, thereby taking care of their homeostasis. Importantly, this mechanism has been shown to increase its activity in HSCs during the regression of fibrosis along with the process of apoptosis. Mitophagy has been shown to affect the induction of apoptosis through an increase in Bcl-B protein [76]. HSC aging, on the other hand, is a physiological and irreversible cellular process. This action naturally contributes to the clearance of myofibroblasts. As a result of telomere shortening, DNA damage, oncogene activation, or the action of oxidative stress, cell cycle arrest occurs. Consequently, cell proliferation stops [51,77]. Aging myofibroblasts induce fibrosis regression through senescence-associated secretory phenotype (SASP). In the process of inducing ECM-degrading enzymes and reducing their proteins, they prevent the continuation of fibrogenic cell formation. NK cells are also involved in this process [71]. ECM degradation is one of the main processes required during fibrosis regression. MMP activation and the downregulation of MMP inhibitory molecules are required for degradation [78]. Aging inducers are also involved in the aging process of active HSCs, which include insulin-like growth factor I (IGF-I) and interleukin 10 (IL-10), interleukin 22 (IL-22), as well as the CCN1/CYR61 stem protein. But aging is also stimulated by drugs, such as celecoxib derivatives and nuclear receptor agonists [79,80]. It has been shown that active HSCs can be inactivated in the process of liver regression [81]. Song et al. showed that the expression of the transcription factors FOXA3, GATA4, HNF1A, and HNF4A causes mouse myofibroblasts to differentiate into hepatocyte-like cells. This study was performed in vivo and in vitro. The results showed the alleviation of liver fibrosis [82]. Another transcription factor 21-Tcf21-which causes the inactivation of HSCs has also recently been detected [83]. Inactivated HSCs are characterized by a novel phenotype. In addition, they show reduced fibrogenic genes such as lysyl Oxidase (LOX), collagens, and α-smooth muscle actin. In addition, they show an increased expression of adipogenic genes that are associated with rest, such as peroxisome proliferator-activated receptor gamma (PPARY-γ) [71]. Not only do HSCs contribute to the myofibroblast population, but also to a lesser extent portal fibroblasts, fibrocytes, but also possibly parenchymal cells undergoing epithelial–mesenchymal transition (EMT) [84].

In addition to myofibroblast reduction, macrophage conversion is important in the regression process. Macrophages are responsible for both damage and repair. The reduction in macrophages in a transgenic mouse model of advanced liver fibrosis led to a reduction in scarring and myofibroblasts [85]. Importantly, during liver regeneration, macrophages change their phenotype. As a result of this process, they stop producing inflammatory and fibrogenic factors and start producing matrix metalloproteases (MMPs). These include MMP9 and MMP12. Importantly, MMPs are enzymes that are capable of devastating the ECM. Both macrophage conversion and MMP induction lead to the phagocytosis of existing ECM. The conclusion is that complete macrophage depletion can disrupt ECM degradation [74]. In addition, myofibroblasts are a source of the inhibitors of metalloproteinase (TIMP). The disappearance of myofibroblasts will result in a reduction in TIMP. In turn, TIMP may contribute to the increased production of MMPs, thereby degrading ECM [86].

Inflammation plays a key role during the fibrogenic process, but also during regression. The inflammatory response includes multicellular interactions that are constantly controlled by multiple factors, e.g., ECM components, soluble mediators, or DAMPs. These multicellular interactions/interactions aim to restore liver function and rebuild the liver architecture. However, when the causative factor persists for too long—it leads to liver fibrosis [74]. One of the promoters of inflammation is programmed cell death. This process induces the secretion of pro-inflammatory and profibrogenic cytokines. These, in turn, cause the activation of HSCs [87]. In addition, the impaired hepatocyte secretes DAMP, which also affects HSC activation through IL-13 (interleukin 13) induction [88]. Inflammatory mediators interact at high levels with activated HSCs. As a result, activated HSCs secrete cytokines or chemokines. These, in turn, can act in a paracrine and autocrine manner. Inflammatory signals in inactivated HSCs affect the activation state (e.g., chemokines) or the maintenance of survival, e.g., interleukin-1 beta (IL-1β), tumor necrosis factor α (TNF-α) through which they are stimuli for inactivated HSCs, but also for inflammatory cells, e.g., CCL2, CCL5. Importantly, the above processes can contribute to both liver progression and regression [74,89].

In their study, Yue et al. showed that Notch signaling was associated with liver fibrosis. It is activated in fibrosis progression, whereas it is inhibited in regression [90]. Mabire et al. used liver sections from patients with end-stage fibrosis and mouse models in their study. They investigated the consequence of inhibiting membrane-associated invariant T cells (MAIT). They found that silencing MAIT slowed the progression of liver fibrosis and even caused regression. The authors suggest that MAIT cells may be one of the targets of antifibrogenic therapy [91].

**Figure 1:**
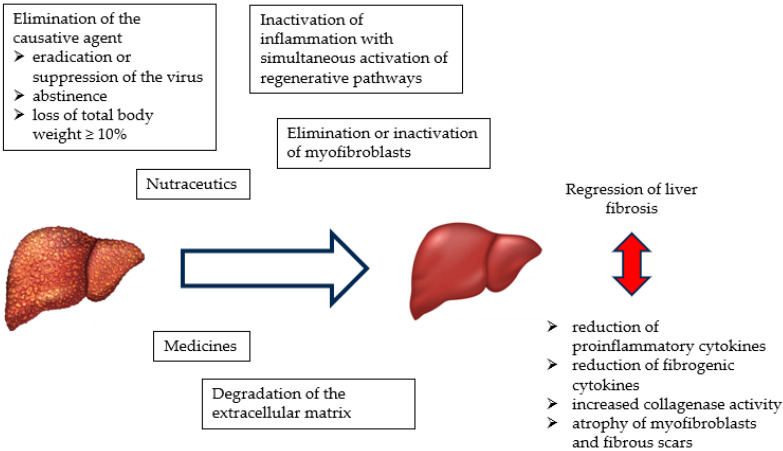
Mechanism of liver fibrosis regression.

A diagram of the mechanisms is shown in Figure 1.

## 4. The Role of Selected Nutraceuticals in Liver Fibrosis

### 4.1. Vitamin C

It has been proposed that vitamin C, also known as ascorbic acid, could potentially inhibit fibrosis and hence prevent liver dysfunction. However, there is currently a lack of data elucidating the precise mechanism through which vitamin C deficiency could contribute to liver fibrosis [92]. Vitamin C plays a crucial role in various essential biological processes within the body, serving as a cofactor for a group of biosynthetic and gene-regulating enzymes, monooxygenases, and dioxygenases. Vitamin C is involved in collagen and carnitine synthesis, as well as the production of catecholamine and amidated peptide hormones. The exact functions are discussed in the publication by Carr and Maginni [93].

**Figure 2:**
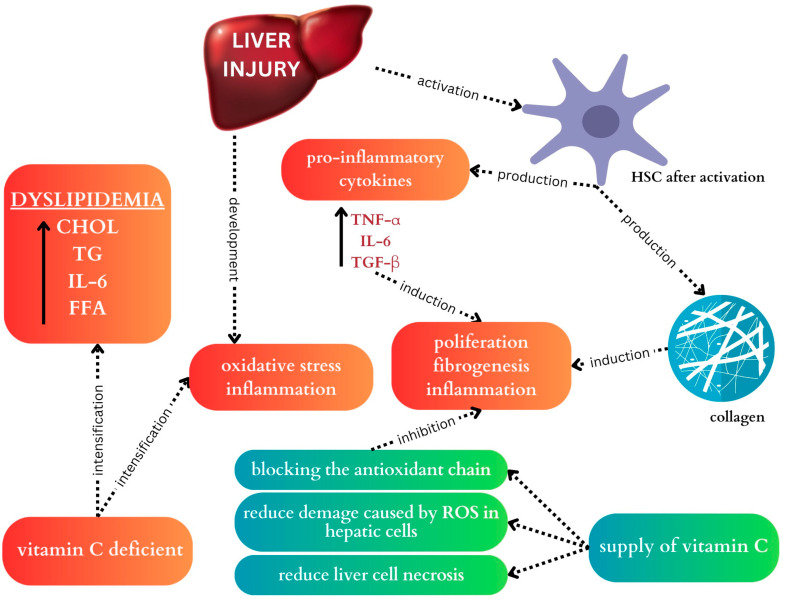
Potential role of vitamin C in the development and prevention of fibrosis. The activation of HSCs may promote the progression of MASLD by secreting TNF-α, IL-6, TGF-β, and collagen, which induces inflammation and fibrosis. Vitamin C deficiency may increase dyslipidemia, oxidative stress, and systemic inflammation. However, an adequate supply of vitamin C may protect against liver cell damage caused by reactive oxygen species and reduce liver cell necrosis as well.

Vitamin C deficiency may exacerbate dyslipidemia, systemic inflammation, and oxidative stress. The elevated levels of inflammation and oxidative stress can trigger the activation of HSC and KCs in the liver. This activation is accompanied by an increased release of TNF-α, IL-6, TGF-β, and collagen, which can lead to cellular damage and fibrosis formation (Figure 2) [94,95].

Liver fibrosis is characterized by the accumulation of ECM proteins in the liver, including collagen and fibronectin. ECMs are essential for the repair of damaged liver tissue; however, if the source of damage is not removed, HSCs continuously activate ECMs, which are deposited and cause liver fibrosis [95]. As mentioned earlier, vitamin C is indispensable for collagen synthesis, acting as a cofactor in the hydroxylation of lysine and proline residues during collagen formation [93,96]. Therefore, its deficiency may disrupt the proper stability and secretion of collagen in the body [95]. On the one hand, ascorbic acid functions as a potent antioxidant and has been suggested to possess antifibrotic properties [7]. On the other hand, it may have a fibrosis-promoting effect by influencing stellate cells. Human HSCs possess only one specialized ascorbic acid transporter, SLC23A2/SVCT2, which is upregulated in cirrhotic patients, whereas human hepatocytes express both SLC23A1/SVCT1 and SLC23A2/SVCT2. It is likely that the use of the selective inhibitors of the SLC23A2 transporter or hydroxylase activity in hepatic stellate cells may bring results in the treatment of liver fibrosis. Recognizing the crucial role of ascorbic acid in the secretion of type I collagen by human HSCs, the modulation of its access or activity represents a potential therapeutic avenue in the context of liver fibrosis [95].

The analysis of data from the National Health and Nutrition Examination Survey (NHANES) showed a relationship between serum vitamin C concentration and a lower risk of developing liver fibrosis in men and overweight or obese patients with MASLD [97]. The previous analysis of NHNES data provided similar evidence. Higher serum vitamin C concentration was inversely associated with the occurrence of metabolic dysfunction, MASLD, and a lower risk of liver fibrosis and cirrhosis. Interestingly, the study participants with obesity and diabetes had lower serum vitamin C levels [98]. Coelho et al. reported serum vitamin C deficiency in 27% of the patients with MASLD. However, no difference was observed in vitamin C concentration in relation to the degree of fibrosis. Importantly, almost half of the respondents provided vitamin C at a level below the estimated average requirement [99]. Results from clinical trials suggest that vitamin C supplementation may have a protective effect on the liver (Table 1) [100,101,102]. A randomized controlled trial by Barbakdze et al. showed that supplementation with vitamin C at a dose of 500 mg/day combined with vitamin E at a dose of 800 mg/day may be useful in reducing damage caused by oxidative stress and slowing the process leading to liver cirrhosis [100]. Interestingly, a previous 6-month study showed that the patients on vitamin C and E therapy (1000 IU and 1000 mg per day) showed improvement in fibrosis even without significant weight loss [101]. The benefits of vitamin E supplementation at a dose of 800 IU/day were also noted by Vilar-Gomez et al. It was observed that vitamin E intake was associated with a significant reduction in overall mortality and hepatic decompensation in patients with or without diabetes with bridging fibrosis and cirrhosis due to MASH [103]. A 2020 systematic review by Abdel-Mabuda et al. showed that vitamin E effectively improves the concentration of liver enzymes alanine aminotransferase (ALT) and aspartate aminotransferase (AST) and MAFLD activity score. Moreover, the authors noted its positive effect in reducing fibrosis in both short- and long-term follow-up in the population of children and adolescents [104]. Interesting observations regarding the reduction in fibrosis were reported by Panera et al. Probably the combination of vitamin E with hydroxytyrosol (HTX), which is a phenolic component of extra virgin olive oil, can reduce TGF-β-induced HSC activation. The combination of these two components was effective, among other things, in reducing the expression of α-smooth muscle actin-α-SMA, which is responsible for increasing ECM stiffness [105].

It is worth paying attention to the fact that vitamin C is necessary to restore vitamin E to its reduced state after its contact with peroxide radicals [106]. It is probable that vitamin C in combination with vitamin E may exhibit stronger anti-inflammatory effects, although the data are still inconclusive. Some studies suggest that such a combination could lead to a decrease in serum reactive C protein (CRP) levels, but these results were only observed in individuals >30 years old, despite earlier observations indicating that they may significantly reduce CRP levels, and these vitamins are characterized by significant total antioxidant capacity [107,108]. In the study by Wajcman et al., the low dietary intake of vitamin C was associated with higher levels of CRP [6]. Fouladvand et al. draw attention to the fact that the ability of antioxidants to reduce pro-inflammatory cytokines may be influenced by many different factors, such as age or fat tissue content [107].

A recent study assessed the association between vitamin C intake and the biomarkers of liver function (total protein, albumin, and alanine aminotransferase). It has been shown that a higher dietary intake of vitamin C was associated with higher albumin levels, which may contribute to improved liver function [109]. Changes in albumin concentration are closely related to the progression of liver diseases and the occurrence of various events, including gastroesophageal varices, liver cirrhosis, and encephalopathy as a consequence of fibrosis [110]. Liver cirrhosis is associated with decreased albumin levels and impaired albumin function [111]. In addition, reduced albumin synthesis may affect calcium transport disorders, and hypocalcemia is associated with a more severe form of decompensated liver cirrhosis [112].

Moreover, it has been demonstrated that vitamin C intake may contribute to reducing ferritin levels, which tend to elevate in patients with MASLD and are correlated with disease severity. Furthermore, based on HbA1c measurements, improved glycemic control has also been observed in this patient group. Therefore, it is suggested that vitamin C may play a significant role in preventing fibrosis in such cases [109]. Vitamin C intake decreased serum ALT, AST, and alkaline phosphatase (ALP) in patients with HCV with elevated liver function test values. Vitamin C probably activates by blocking the antioxidant chain, thereby preventing the negative effects of ROS, which may contribute to cell membrane damage in liver cells [113]. In the study by He et al., vitamin C supplementation in the amount of 1000 mg/day also significantly reduced the concentration of AST and ALT in serum [102].

### 4.2. Omega-3 Polyunsaturated Fatty Acids (PUFAs)

Omega-3 PUFA acids have long been the subject of research on their use in both the prevention and treatment of many diseases such as cardiovascular diseases, diabetes, neurodegenerative diseases, and cancer [114]. Omega-3 acids play an important role in the body as the components of phospholipids that create the structures of cell membranes, are used to create eicosanoids, are involved in the release of neurotransmitters, and are involved in gene expression. They also have strong anti-inflammatory and immunomodulatory effects [115].

In patients with MASLD, an association between liver fatty acid composition and fibrosis is observed. Fridena et al. reported a positive relationship between arachidic acid and an inverse relationship between docosahexaenoic acid (DHA), oleic acid, and oleic acid combined with vaccenic acid and liver fibrosis. Furthermore, DHA, a biomarker of oily fish intake, was inversely associated with liver fibrosis [116]. A meta-analysis of studies conducted by He et al. showed that n-3 supplementation may have a beneficial effect on reducing the concentration of triglycerides (TG), ALT, total cholesterol (TC), and high-density lipoprotein cholesterol (HDL-C) in serum in patients with MASLD. Omega-3 PUFAs also tend to have a beneficial effect on AST, gamma-glutamyltranspeptidase (GGT), and low-density lipoprotein cholesterol (LDL-C) levels [117].

The effect of n-3 supplementation still remains unclear in terms of liver fibrosis due to limited data, some of which comes from studies in animal models. Nevertheless, results from studies in animal models confirm their immunomodulatory and anti-inflammatory effects on liver diseases and their important role in reducing liver fibrosis by improving its regeneration [118,119]. The supply of fish oil to a mouse model has been shown to reduce CCl4-induced liver fibrosis. Additionally, n-3 fatty acids decrease the expression of genes that promote fibrosis in both activated liver cells responsible for scarring (HSCs) and in the liver affected by fibrosis. This suppression of gene expression is controlled by a protein called YAP, making YAP a specific target of n-3 fatty acids. Furthermore, the researchers illustrated that in fibrotic livers and activated HSCs, there is an excess of YAP/TAZ proteins, which are then broken down with the help of n-3 fatty acids in a process dependent on proteasomes [120].

**Figure 3:**
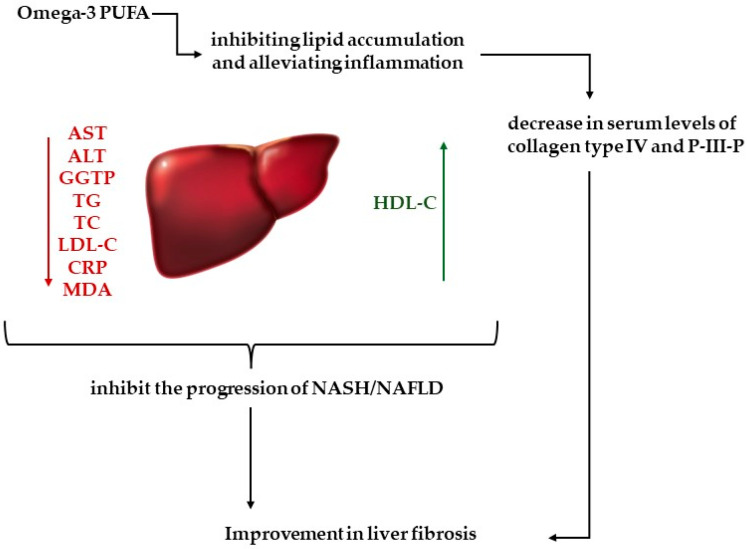
Potential effect of omega-3 PUFA on liver fibrosis [97,98,99]. Omega-3 PUFA may improve parameters such as AST (aspartate aminotransferase), ALT (alanine aminotransferase), GGTP (gamma-glutamyl transpeptidase), TG (triglycerides), TC (total cholesterol), LDL-C (low-density lipoprotein cholesterol), CRP (C-reactive protein), MDA (malondialdehyde), and HDL-C (high-density lipoprotein cholesterol). They inhibit the accumulation of lipids, alleviate inflammation, and reduce the level of collagen type IV and P-III-P in the serum.

Studies involving humans also show promising results (Table 2). An analysis of British Biobank data by Vell et al. showed that the regular consumption of n-3 fatty acids was associated with a significant reduction in the risk of developing liver disease, especially in the case of MASLD [121]. In a study by Cansanção et al., oral n-3 supplementation in the form of fish oil capsules (503 mg DHA and 103 mg eicosapentaenoic acid (EPA)) three times daily for 6 months showed a significant reduction in liver fibrosis. Additionally, a reduction in waist circumference, gamma-glutamyl transferase, TC, TG, and a controlled attenuation parameter was observed [122]. Similar results were observed by Li et al. The supply of n-3 (50 mL PUFA in a 1:1 ratio of EHA and DHA) also over a period of 6 months improved parameters such as ALT, AST, TG, CRP, malondialdehyde (MDA) as well as collagen type IV and P-III-P. This means that it is possible to inhibit the progression of MASH as a result of n-3 supply by alleviating hepatic inflammation and oxidative. In turn, a decrease in collagen suggests an improvement in liver fibrosis (Figure 3) [123].

An interesting observation is provided by a recent meta-analysis of studies conducted by Padiadpu et al., which showed that the reduction of betacellulin (BTC), an epidermal growth factor receptor ligand, by DHA may potentially prevent/treat fibrosis. This is because BTC induces TGFβ-2, a key contributor to liver fibrosis through collagen production by hepatic stellate cells. Moreover, BTC improves pathways related to the function of mitochondria, the function of which may be impaired in the course of MASLD/MASH, and interacts with microbiological signals in the induction of integrins [124].

Different results were obtained by Argo et al. n-3 supplementation at a dose of 3000 mg/day for one year did not lead to significant changes in overall histological activity in patients with MASH (primary end point: reduction in NAS by ≥2 points). Omega-3 therapy was associated with a reduction in liver fat, independent of body weight loss [125]. Similar observations regarding the reduction in fat content in the liver were observed by Scorletti et al.; however, no improvement in the reduction in fibrosis was noted [126]. In a study by Sanyal et al., which assessed various doses of EPA supplementation in patients with MASLD and MASH, the high doses of EPA of 2700 mg/d only reduced TG levels. However, there was no effect on the degree of steatosis, inflammation, ballooning, or fibrosis [127].

**Table 2:** Clinical studies discussed in the review regarding omega-3 PUFA supplementation and its effect on liver performance.

Population	Intervention Duration	Methods	Results	Reference
Patients diagnosed with MASLD n = 24 Randomly assigned to 2 groups: Study group n = 13 Control group n = 11	6 months	Patients received 503 mg DHA and 103 mg EPA 3 times daily or placebo.	Docosahexaenoic acid (DHA) and omega index increased significantly in RBC in addition to a significant reduction in alkaline phosphatase (ALP) and liver fibrosis. There has been a reduction in ALP and liver stiffness measure (LSM) as measured by FibroScan^®^ Reductions in waist circumference (WC), gamma-glutamyl transferase (GGT), total cholesterol (CT), triglycerides (TG), and controlled attenuation parameter (CAP) were noted, although statistical significance was not attained. No significant changes in the assessed parameters were observed in the placebo group. There was no statistically significant difference in the relative expression of miR-122 when comparing the baseline period to the six-month intervention, observed in both the placebo group and the n-3 PUFA group.	Cansanção et al. 2020 [122]
Patients diagnosed with MASH n = 78 Randomly assigned to 2 groups: Study group n = 39 Control group n = 39	6 months	Patients received 50 mL of PUFA in a 1:1 ratio of EHA and DHA in their daily diet). Additionally, both groups were recommended to engage in moderate physical exercise lasting 30 min at least 5 days a week. A low-fat, low-cholesterol, and low-carbohydrate diet was also recommended.	There was an improvement in parameters such as AST (aspartate aminotransferase), ALT (alanine aminotransferase), GGTP (gamma-glutamyl transpeptidase), reactive C protein (CRP), malondialdehyde (MDA) as well as collagen type IV and P-III-P. Positive results were obtained in terms of steatosis grade, necrotic-inflammatory grade, fibrosis grade, and ballooning results compared to the control group. Histological characteristics were comparable between the control group and the PUFA-treated group.	Li et al. 2015 [123]
Patients diagnosed with MASH n = 34 Randomly assigned to 2 groups: Study group n = 17 Control group n = 17	12 months	Patients received n-3 at a dose of 3000 mg/day with advice on 150 min of physical activity per week and a hypocaloric diet (the reduction of 30% of daily kcal intake).	There was a greater decrease in liver fat content. Supplementation did not lead to improvement in the primary histological activity score in patients with NASH (≥2-point reduction in NAS). No independent effects on the markers of hepatocyte damage or the indicators of insulin sensitivity were observed. There was no improvement in cell injury biomarkers (M30 and M65) that did not occur uniformly with N-3 PUFA and only occurred with concomitant weight loss. There were no consistent beneficial effects on blood lipid composition (aside from a trend in triglyceride reduction) or insulin sensitivity.	Argo et al. 2015 [125]
Patients diagnosed with MASLD n = 103 Randomly assigned to 2 groups: Study group n = 51 Control group n = 52	15–18 months	Patients received 4 g/day of DHA + EPA (460 mg EPA and 380 mg DHA) or placebo.	There was a trend toward improvement in liver fat percentage. DHA erythrocyte enrichment was independently associated with a decrease in liver fat percentage. No improvement in fibrosis scores occurred.	Scorletii et al. 2014 [126]

### 4.3. Carotenoids

Carotenoids to a group of fat-soluble compounds. They are plant pigments that have a highly antioxidant effect by scavenging free radicals [128]. β-Carotene is a precursor to vitamin A. Moreover, it has a higher potential for this vitamin compared to α-carotene or β-cryptoxanthin [129,130]. In addition, β-carotene is the most common carotenoid in the liver [131]. Other sites of its reserves include muscles, kidneys, skin, but also adrenal glands and mammary glands [132]. Its dietary sources include tomatoes, red watermelon, mango papaya, pumpkin, and pineapple [133].

In the study, Chaves et al. showed that of 145 patients diagnosed with grade III obesity (BMI ≥ 40 kg/m^2^), as many as 71% had comorbid MASLD. These patients had significantly lower serum β-carotene levels compared to the group without MASLD. The authors suggest that this is due to an increased need for vitamin A due to oxidative stress. In addition, there is a link in the study which shows that β-carotene deficiency could potentially correlate with insulin resistance comorbidity [134]. In addition, there are studies showing that β-carotene has damage-reducing effects on the liver. Martin et al. demonstrated in vivo that β-carotene has a protective effect by preventing tert-butyl hydroperoxide-induced oxidative damage in HepG2 cells [135]. In contrast, Seifert et al. examined β-carotene supplementation in rats with CCl4-induced liver damage. The results showed a reduction in liver hydroxyproline content and fibrosis compared to a control group of rats that received no supplementation [136]. Another study involving rats also showed similar results. In rats with induced cirrhosis by thioacetamide, it was observed that β-carotene supplementation could reduce liver fibrosis [137]. A similar study was performed by El-baz et al. who used an extract of Haematococcus pluvialis, which is rich in β-carotene, among other things. The results showed that Haematococcus pluvialis caused a reduction in the concentrations of liver enzymes, collagen 1, nitric oxide, as well as α-smooth muscle actin and TGF-β. In addition, the extract affected the balance between metalloproteinase and its inhibitor, induced KC proliferation, and inhibited liver fibrosis. The authors conclude that Haematococcus pluvialis has an effect on the regression of fibrosis by several mechanisms. These include the regulation of inflammatory mediators, regulation of oxidative stress, inhibition of profibrogenic factors, or modulation of metalloproteinase [138]. In a study on rats in which liver fibrosis was artificially induced, the effects of the β-carotene-rich marine phytoplankton, *Dunaliella salina*, were examined. It turned out that *Dunaliella salina* influenced a significant decrease in ASPT, ALT, bilirubin, and MDA. In addition, histopathological results showed improved fibrosis and reduced inflammatory cell infiltration [139]. In turn, Ozturk et al. showed that rats’ consumption of feed containing apricots, which are abundant in β-carotene, reduced serum ALT and AST levels, but also reduced liver damage [140]. In addition, it appears that β-carotene can inhibit HCV replication in the cell culture system [141]. 9-cis β-carotene as an isomer of β-carotene has been shown to reduce cholesterol and atherosclerosis, but also inhibits inflammation in the liver of mice [142]. In an experimental study on a rat model of induced MASH, the administration of an herbal derivative, Lycium barbarum polysaccharides, was used. Lycium barbarum, rich in β-carotene, was found to have hepatoprotective properties by affecting the alleviation of fibrosis and oxidative stress [143]. The main objective of the study by Sandoval et al. was to determine whether the oral administration of β-carotene would affect liver biochemical characteristics in rats that had been exposed to an ethanol supply. It was shown that low doses could show beneficial effects on liver damage and prevent liver steatosis during alcohol consumption. However, the authors point out, in order to avoid the more serious effects of alcohol consumption, other factors should be taken into account, such as the amount of alcohol consumed, the time of exposure, but also the mechanisms responsible for regulating alcoholic liver disease [144]. The complex repair effect of β-carotene was also demonstrated in the liver of mice in which damage was induced by Angiotensin II administration. The damage repair effect was caused by controlling Kcs, monocytes, and inflammatory macrophages, but also controlling the mediator of the plasminogen activator system [145]. Liu et al. in a study involving 4352 people showed that higher β-carotene intake was inversely related to liver steatosis [146]. In an observational study on 72 patients with MASLD, fibrosis levels were measured using FibroScan. In addition, serum retinol, alpha-tocopherol, ascorbic acid, beta-carotene, and selenium were assessed, but data on the intake of these micronutrients were also collected. The study found a prevalence of retinol, selenium, and vitamin C deficiency, but also a frequent inadequate intake of vitamin A, vitamin C, vitamin E, selenium, and β-carotene. Importantly, a low intake of β-carotene may have an impact on low serum retinol levels [147]. In a study on 69 people with HCV, it was noted that a decrease in serum β-carotene levels is associated with the early phase of the disease. Moreover, it was shown that the process of vitamin A loss is accompanied by the activation of stellate cells [148].

Other noteworthy carotenoids include lycopene and astaxanthin, which also have high antioxidant and anti-inflammatory effects. Lycopene is a pigment found mainly in tomatoes, but also in apricots, melons, papayas, and peaches. Its biological activity is based on antioxidant, anticancer, cardioprotective, neurobiological, anti-inflammatory, and anti-aggregation effects [149]. Astaxanthin, on the other hand, is an orange to dark red color pigment that can be found in marine organisms. It has recently received a lot of attention for its wide-ranging effects [150].

Gao et al. showed that in mice on high-fat and high-fructose diets, lycopene supplementation has the potential to reduce the risk of MASLD. This appears to be due to the inhibition of the NF-κB/NLRP3 inflammasome pathway, but also through beneficial effects on the gut microbiota [151]. In addition, similar results, but with respect to liver fibrosis, were found by Li et al. They showed that lycopene can cause the regression of fibrosis in male rats in which liver fibrosis was induced by the application of carbon tetrachloride. Potential mechanisms for this effect include effects on reducing oxidative stress and inflammation [152]. Ni et al. in their study on mice with induced MASH showed that lycopene administration reduces lipid accumulation in the liver and increases lipolysis [153]. An additional advantage of lycopene is its possible potential in the treatment of obesity and metabolic syndrome, which are associated with MAFLD and liver fibrosis [154]. Astaxanthin is shown to be more effective than vitamin E in preventing lipid peroxidation. In addition, it is shown to have hepatoprotective and anti-inflammatory properties. In addition, it has a protective effect against neurodegeneration, cardiovascular disease, and diabetes [155,156]. In mice with induced NAH, astaxanthin was found to reduce peroxidation and lipid accumulation in the liver. In addition, by reducing CD4 and CD8 T-cell recruitment, it reduced inflammation and insulin resistance. Importantly, it potentially reverses fibrosis in the early stages of MASH [157]. It has been shown that astaxanthin may have antifibrotic effects as a result of blocking TGFβ1 signaling, with the consequent activation of the Smad3 pathway in HSCs. This, in turn, may inhibit fibrogenic gene expression [158]. In addition, it is possible that astaxanthin may have an effect on reducing the activation of HSC cells, which play one of the main roles in the induction of fibrosis [159]. In addition, Islam et al. showed that in mice with CCl4-induced liver damage, astaxanthin has a hepatoprotective effect. This is likely due to the stimulation of the immune system and the reduction in lipid peroxidation [160]. Ultimately, however, the hepatology societies require long-term randomized clinical trials that will ultimately draw conclusions on the guidelines for carotenoid use [9].

### 4.4. Curcumin

Curcuma longa is a rhizome that has been known since ancient times and can come in 70 varieties [161]. It contains curcumin, an active compound that is extracted from its rhizome. It belongs to polyphenolic hydrophobic compounds. It shows a wide spectrum of activity in the food and textile industries due to its yellow color [162]. The main compounds with bioactive effects are essential oils and curcuminoids [163]. Turmeric is easily degraded and has low bioavailability after oral administration, so various formulations are being developed to improve absorption by the body [164,165]. It is metabolized in the intestinal mucosa and in the liver [166]. Akter et al. showed that antioxidant properties, through the content of phenols and flavonoids, vary depending on the variety of turmeric. The Ryudai gold variety contained the highest concentration of antioxidant substances [167].

The main compounds that have been isolated from turmeric are as follows:

○ Curcumin;
○ Demethoxycurcumin;
○ Bisdemethoxycurcumin;
○ Cyclocurcumin.

Curcuma oils are often presented by researchers as substances that support the immune system, promote the excretion of toxins from the body, and support the digestive process [168]. Due to its various properties, curcumin has shown beneficial effects on various parts of the digestive tract.

**Figure 4:**
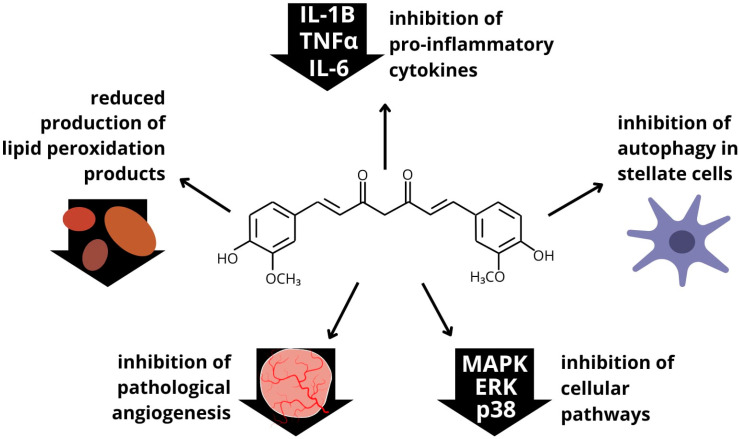
Effect of curcumin on the healing process of liver fibrosis.

Kong et al. in their work presented that curcumin, due to its antioxidant properties, by inhibiting the increase in the level of reactive oxygen species (ROS) and regulating the process of autophagy, which plays an important role in the pathogenesis of liver fibrosis, can attenuate the epithelial–mesenchymal transition, thus exhibiting antifibrotic effects [169]. In addition, in another paper, the authors indicate that curcumin exhibits not only a protective effect on the organ but also a therapeutic effect. This type of action is achieved by modulating the signaling of various cellular pathways, e.g., ERK/p38/MAPK. Furthermore, it can reduce the production of lipid peroxidation products and inhibit the production of pro-inflammatory cytokines (IL-1β, TNFα, and IL-6) [170]. Therefore, Ma et al. in their study evaluated the therapeutic efficacy of the curcumin analog L6H4. The study was carried out in animal models, in which they observed, after eight weeks, a significant improvement in organ structure, so it may exhibit properties to alleviate liver fibrosis [171]. Another mechanism of the action of curcumin is the attenuation of TGFβ1 signaling [172]. The substance may also reduce the level of the activation of liver stellate cells, as well as their migration, by inhibiting the biological axis CXCL12/CXCR4 [173]. The inhibition of pathological angiogenesis can also occur through curcumin’s effects on the mTOR, ERK, and FAK/RhoA signaling, but PPAR-γ must first be activated to have this effect [174]. By suppressing activated stellate cell autophagy, the substance can inhibit its activity and induce apoptosis [175]. Another mechanism of the antifibrotic effect of curcumin on the liver is the demethylation of certain genes [176]. As indicated by Chan et al., this compound that exhibits bioactive activity may be the basis for the development of new drugs, the basis of which may be a molecular mechanism based on the action of curcumin, for example, to assist in the treatment of liver fibrosis [177]. A summary of the effect of curcumin on liver fibrosis is shown in Figure 4.

## 5. Conclusions

The process of liver fibrosis has a complex mechanism in which there is altered signaling of certain processes that cause changes in organ healing and the initiation of fibrinogenesis. In recent years, research has focused on the phenomenon of the regression of fibrosis, which was once thought to be irreversible. In particular, nutraceuticals are attracting the attention of researchers, which in the future could potentially aid in the treatment of patients with chronic liver disease (MASLD/MASH). One substance with potential therapeutic effects in liver fibrosis is curcumin, which exhibits bioactive activity, which could be the basis for developing new drugs with this compound to support the healing process. Supplementation with vitamin C, beta-carotene, and omega-3 fatty acids in patients with liver damage caused by oxidative damage resulted in an improved liver function and other metabolic parameters related to glucose and fatty acid metabolism in the body. These compounds may be an adjunctive therapy option for both MASLD and MASH patients, and even in the process of organ fibrosis. However, there is still a need for long-term, randomized, controlled clinical trials to accurately assess whether these dietary compounds can completely reverse fibrosis and reduce the incidence of liver failure. 

## Figures and Tables

**Table 1:** Clinical studies discussed in the review regarding vitamin C supplementation and its effect on liver performance.

Population	Intervention Duration	Methods	Results	Reference
Patients diagnosed with MASH n = 107 Randomly assigned to 3 groups: Study group n = 35 n = 52 Control group n = 20	12 months	Patients were randomly assigned to receive ursodeoxycholic acid (UDCA) 15 mg/kg/day (group A) or vitamin E 800 mg/day plus vitamin C 500 mg/day (group B) and a control group that did not receive any treatment.	After 12 months of treatment with vitamins E and C, compared to UDCA, a significant reduction in the average level of alanine aminotransferase (ALT) was observed. There was a reduction in both the mean steatosis score and fibrosis score.	Barbakadze et al. 2019 [100].
Patients diagnosed with MASH n = 49 Randomly assigned to 2 groups: Study group n = 23 Control group n = 22	6 months	Patients randomized to receive vitamins E and C (1000 IU and 1000 mg, respectively) or placebo daily.	Significant improvement in fibrosis was noted within the group that received vitamins E and C but not in the placebo group. No statistically significant difference in fibrosis was noted between the vitamin and placebo groups. There were no changes in the ALT concentration in the study group and no differences in the AST value between the groups. The evaluation of the histologic data demonstrated no statistically significant differences in inflammation/necrosis score.	Harrisona et al. 2003 [101]
Patients diagnosed with MASLD n = 24 Randomly assigned to 3 groups with different vitamin C intakes: Study group n = 26 n = 30 n = 28	12 weeks	Patients treated with low (250 mg/day, n = 26) or medium (1000 mg/day, n = 30) or high (2000 mg/day, n = 28) doses of oral vitamin C supplements.	In the medium supply group, a more significant decrease in the concentration of AST and ALP was observed compared to the high supply group. There was no statistically significant difference in ALT or AST between the low- and high-dose vitamin C groups. Liver health indicators such as gamma-glutamyltransferase, alkaline phosphatase, total bilirubin, direct bilirubin, and glucose metabolism parameters such as fasting insulin, fasting glucose, and homeostasis model assessment for insulin resistance decreased after the intervention but were comparable in the three groups.	He et al. 2021 [102]
